# Supplementary material for: Moving towards malaria elimination in southern Mozambique: Cost and cost-effectiveness of mass drug administration combined with intensified malaria control
Source: PLoS One. 2020 Jul 6;15(7):e0235631. doi: 10.1371/journal.pone.0235631 (PMC7337313; doi:10.1371/journal.pone.0235631)
Supplement: S1 Fig — (DOCX) [file pone.0235631.s001.docx]

**Figure S1. ICER evolution ("direct evidence Magude project" and "governmental perspective")**

This figure plots the deterministic incremental cost-effectiveness ratio of the Magude project (versus routine malaria control) at three different timepoints (by end year 1, year 2 and year 3). It also shows the ICER evolution if activities were implemented from a governmental perspective (see S6 Table for details). The horizontal blue lines represent the standard high cost-effectiveness threshold equal to one time the gross domestic product per capita ($468 per DALY averted) and the standard cost-effectiveness threshold equal to three time the gross domestic product per capita ($1,404 per DALY averted). DALYs=disability adjusted life-years.
