# Supplementary material for: Moving towards malaria elimination in southern Mozambique: Cost and cost-effectiveness of mass drug administration combined with intensified malaria control
Source: PLoS One. 2020 Jul 6;15(7):e0235631. doi: 10.1371/journal.pone.0235631 (PMC7337313; doi:10.1371/journal.pone.0235631)
Supplement: S2 Fig — (DOCX) [file pone.0235631.s002.docx]

**Figure S2. Cumulative malaria cases averted across time (2015-2018)**

This figure plots the cumulative number of averted malaria cases by the Magude project at three different timepoints (by end year 1, year 2 and year 3), which amounted to 38,369 by end year 3 (June 2018). Further details on the impact evaluation study design, methods and results are provided elsewhere (Galatas et al, 2020)
