# Supplementary material for: Moving towards malaria elimination in southern Mozambique: Cost and cost-effectiveness of mass drug administration combined with intensified malaria control
Source: PLoS One. 2020 Jul 6;15(7):e0235631. doi: 10.1371/journal.pone.0235631 (PMC7337313; doi:10.1371/journal.pone.0235631)
Supplement: S3 Fig — (DOCX) [file pone.0235631.s003.docx]

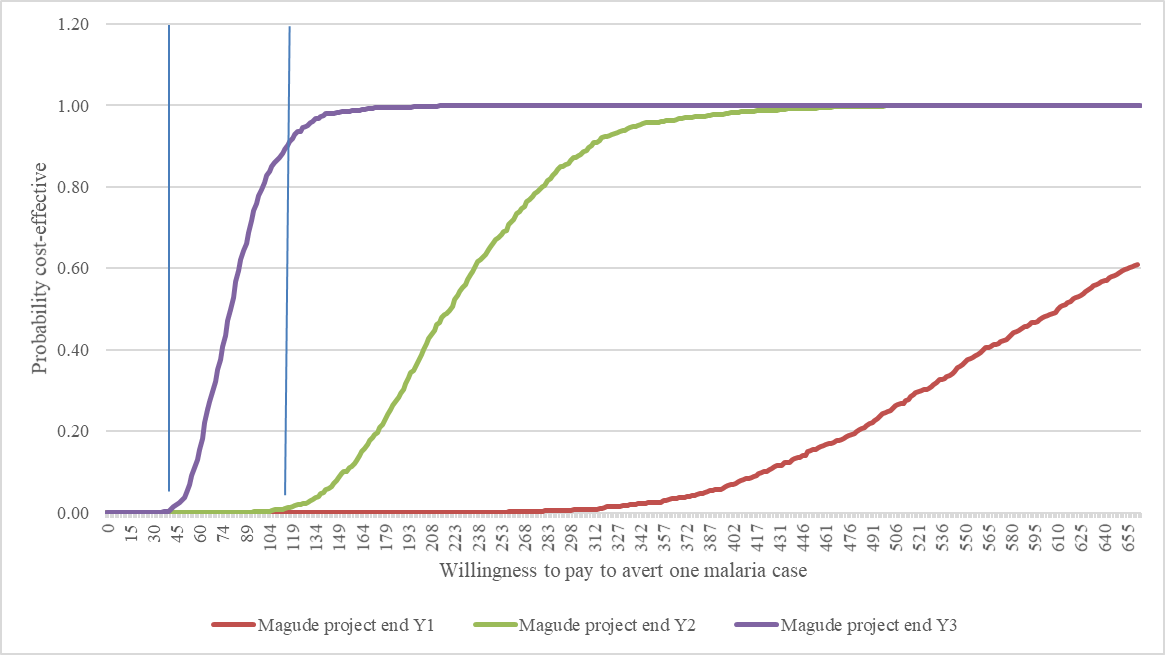


**Figure S3. Cost-effectiveness acceptability curve per malaria case averted**

The acceptability curves show the probability that the Magude project is cost-effective (compared to routine malaria control) across time (by end year 1, year 2 and year 3) for different levels of willingness to pay to avert one malaria case (X axis). The vertical lines represent critical WTP per malaria case averted that can be applied to Mozambique: US$39 per malaria case averted (standard threshold of highly cost-effective interventions; and US$116 per malaria case averted (standard threshold of cost-effective interventions)
