# Supplementary material for: Moving towards malaria elimination in southern Mozambique: Cost and cost-effectiveness of mass drug administration combined with intensified malaria control
Source: PLoS One. 2020 Jul 6;15(7):e0235631. doi: 10.1371/journal.pone.0235631 (PMC7337313; doi:10.1371/journal.pone.0235631)
Supplement: S4 Fig — (DOCX) [file pone.0235631.s004.docx]

**Figure S4. Cumulative costs across time, 2015-2030 (US$ million)**

Cumulative costs, between January 2015 and 2030 under three different scenarios: a) the Magude project (i.e. evidence from the pilot project), the Magude project from a governmental perspective (i.e. project implemented in programmatic mode) and the routine malaria control scenario.
