# Supplementary material for: Moving towards malaria elimination in southern Mozambique: Cost and cost-effectiveness of mass drug administration combined with intensified malaria control
Source: PLoS One. 2020 Jul 6;15(7):e0235631. doi: 10.1371/journal.pone.0235631 (PMC7337313; doi:10.1371/journal.pone.0235631)
Supplement: S1 Table — (DOCX) [file pone.0235631.s005.docx]

**S1 Table. Input variables and probabilistic distribution for cost-effectiveness analysis**

**Reference List**

1. WHO. WHO guide to cost-effectiveness analysis. Geneva2003.

2. Arroz JAH, Candrinho B, Pedro S, Mathe G, da Silva M, Tsabete S, et al. Planning and implementation of a countrywide campaign to deliver over 16 million long-lasting insecticidal nets in Mozambique. Malar J. 2018;17(1):254. Epub 2018/07/11. doi: 10.1186/s12936-018-2406-2. PubMed PMID: 29986711; PubMed Central PMCID: PMCPMC6038318.

3. Cico A, Johns B. PMI IRS Country Programs: 2017 Comparative Cost Analysis. 2018.

4. Price and quality reporting [Internet]. The Global Fund. 2019. Available from: <https://www.theglobalfund.org/en/sourcing-management/price-quality-reporting/>.

5. Sicuri E, Bardaji A, Nhampossa T, Maixenchs M, Nhacolo A, Nhalungo D, et al. Cost-effectiveness of intermittent preventive treatment of malaria in pregnancy in southern Mozambique. PLoS One. 2010;5(10):e13407. Epub 2010/10/27. doi: 10.1371/journal.pone.0013407. PubMed PMID: 20976217; PubMed Central PMCID: PMCPMC2955525.

6. The World Bank. The World Bank Open Data 2018.

7. Galatas, B., Saúte, F., Martí-Soler, H., Montañà, J., Guinovart, C., Munguambe, H., . . . Aide, P. (2020). The Magude project: a before-after study aiming to eliminate malaria in southern Mozambique. Manuscript under review (PLos Med).

8. WHO. Global Burden of Diseases 2004 update: disability weights for diseases and conditions 2019 [cited 2019 April, 25]. Available from: <https://www.who.int/healthinfo/global_burden_disease/GBD2004_DisabilityWeights.pdf?ua=1>.
