# Supplementary material for: Moving towards malaria elimination in southern Mozambique: Cost and cost-effectiveness of mass drug administration combined with intensified malaria control
Source: PLoS One. 2020 Jul 6;15(7):e0235631. doi: 10.1371/journal.pone.0235631 (PMC7337313; doi:10.1371/journal.pone.0235631)
Supplement: S4 Table — (DOCX) [file pone.0235631.s008.docx]

**S4 Table. Economic costs per budget category and activity (2015-2018)**
