# Supplementary material for: Moving towards malaria elimination in southern Mozambique: Cost and cost-effectiveness of mass drug administration combined with intensified malaria control
Source: PLoS One. 2020 Jul 6;15(7):e0235631. doi: 10.1371/journal.pone.0235631 (PMC7337313; doi:10.1371/journal.pone.0235631)
Supplement: S6 Table — (DOCX) [file pone.0235631.s010.docx]

**S6 Table. The Magude project costs ("direct evidence Magude project" vs. "governmental perspective")**
