# Supplementary material for: Moving towards malaria elimination in southern Mozambique: Cost and cost-effectiveness of mass drug administration combined with intensified malaria control
Source: PLoS One. 2020 Jul 6;15(7):e0235631. doi: 10.1371/journal.pone.0235631 (PMC7337313; doi:10.1371/journal.pone.0235631)
Supplement: S1 Text — (DOCX) [file pone.0235631.s012.docx]

**S1 Text. Costing formulas**

The formulas defined below describe the methodological approach for estimating the economic costs of the project for each activity *s*. First, we can define the total economic costs (in constant $US 2015) of each malaria intervention with the following formula:

$A_{s}=\sum_{j=1}^{m} \sum_{t_{0}=2015}^{n} \frac{{\left( P_{jt}*Q_{jt} \right)(M*T*I)\left( 1+i \right)}^{(n-t)}}{{L_{jt}*\left( 1+d \right)}^{(n-t)}}$ , where **(1.1)**

- $A_{s}$ is the cost of each malaria elimination/control activity costed, for activities *s=1,2,3,4,5* (i.e. MDA, IRS, epidemiological surveillance, rfMDA or LLIN).
- *j=1,2……m* is the item costed under activity *s* (i.e. salaries, vehicles, per diems for a training, fuel for a supervision visit, etc.)
- *t* is the year of analysis, for *t=2015, 2016, ….n*, and $t_{0}=2015$ is the baseline year
- *(n-*$t_{0}$*)* is the timespan (number of years) from baseline year
- $P_{jt}$ is the unit cost of item *j* at time *t*
- $Q_{jt}$ is the quantity of item *j* at time *t*
- $i$ is the interest rate (% per annum)
- $d$ is the discount rate (% per annum)
- *M* is the maintenance costs, assumed to be 15% of the capital costs. *M=0* if item *j* is a recurrent cost (non-capital)
- *T* is the transport costs from in-country distribution (only applicable to imported good, mainly drugs and commodities), assumed to be 25% of acquisition costs.
- *I* is the importation costs, including freight and insurance (only applicable to imported good, mainly drugs and commodities), assumed to be 40% of acquisition costs.
- $L_{jt}$ is the useful years of life for capital goods, the years used to annualize capital goods

In addition, the case management costs of treating outpatient visits as well as inpatient hospital admissions, were calculated by:

${Inp}_{t}={NInp}_{t}*[Art+Coart+\left( D*A \right)]$ , where **(1.2)**

- ${Inp}_{t}$ is the inpatient costs at time *t*
- *t* is the year of analysis, for *t=2015, 2016, ….n*
- ${NInp}_{t}$ is the number of inpatient admissions at time *t*
- *Art* is the average cost of artesunate (treatment for severe malaria), taking into consideration average dosage and drug price
- *Coart* is the average cost of coartem treatment, taking into consideration average dosage and drug price
- *D* is the average number of days of hospitalization (assumed to be 5).
- *A* is the costs of an admission at the hospital (occupied bed/day)

And by

${Out}_{t}={NOut}_{t}*\left( Coart+R \right)$ , where **(1.3)**

- ${Out}_{t}$ is the outpatient costs at time *t*
- *t* is the year of analysis, for *t=2015, 2016, ….n*
- ${NOut}_{t}$ is the number of outpatient visits at time *t*
- *Coart* is the average cost of coartem treatment for uncomplicated malaria, taking into consideration average dosage and drug price
- *R* is the average recurrent costs of an outpatient visit, consisting of personnel, medical, surgical and laboratory supplies.

Given equations (1.2) and (1.3), the total cost of the illness (COI) across the period of study *(t=2015,…n)* in constant US$ 2015 is defined by:

$COI=\sum_{t_{0}=2015}^{n} \frac{{\left( {Inp}_{t}+{Out}_{t} \right)\left( 1+i \right)}^{(n-t)}}{\left( 1+d \right)^{(n-t)}}$ , where  **(1.4)**

- *COI* is the total cost of illness
- *t* is the year of analysis, for *t=2015, 2016, ….n*
- ${Inp}_{t}$ is the inpatient costs at time *t*
- ${Out}_{t}$ is the outpatient costs at time *t*
- $i$ is the interest rate (% per annum)
- $d$ is the discount rate (% per annum)

Finally, taking into consideration equations (1.1) and (1.4), the total costs of the Magude project in constant US$2015 are defined by:

$Total costs=\sum_{s=1}^{k} A_{s}+ COI$ , where **(1.5)**

- $A_{s}$ is the specific malaria elimination/control activity being costed, for *s=1,2,3,4,5* (i.e. MDA, IRS, epidemiological surveillance, rfMDA or LLIN).
- *COI* is the total cost of illness across the period of study

Formulas (1.1) to (1.5) do also define the methodological approach for calculating the costs under the other scenarios (i.e. Magude project from a governmental perspective and the control scenario). However, for these scenarios, specific costing items, prices, quantities and activities were considered.
