# Supplementary material for: Moving towards malaria elimination in southern Mozambique: Cost and cost-effectiveness of mass drug administration combined with intensified malaria control
Source: PLoS One. 2020 Jul 6;15(7):e0235631. doi: 10.1371/journal.pone.0235631 (PMC7337313; doi:10.1371/journal.pone.0235631)
Supplement: S2 Text — (DOCX) [file pone.0235631.s013.docx]

**S2 Text. Costs projection assumptions (2015-2030)**

We elaborated a costing scenario till 2030 based on the assumption that the gains achieved by the third year could be maintained by continuing with standard vector control approaches targeted to insecticide resistance contexts, together with a strengthened surveillance and a response system using rfMDA, on top of standard case management and LLIN distribution. However, there is still a lot of uncertainty on the optimal combination of strategies to sustain the gains in the study area and further evidence is needed, so results from this exercise are only indicative of a possible costing scenario that might accrue in the future under the Magude project*.*  More specifically:

- *Mass Drug Administration (MDA).* Activities related to MDA were assumed to be implemented in the initial phase of the project (2015-2017) in order to reduce malaria transmission levels to pre-elimination levels but not repeated afterwards.

- *Epidemiological surveillance.* The enhanced surveillance system at the health facilities and community health workers, was assumed to be maintained throughout all the period.
- *Indoor residual spraying (IRS).* Annual rounds of indoor residual spraying were assumed to be in place until 2030. However, universal IRS was assumed to be implemented until 2020 and to be progressively targeted to specific malaria hot-spots areas after that. This assumption translated into a reduction in IRS costs by 20% (from 2020 to 2022) and by 50% (from 2022 to 2030).
- *Reactive focal mass drug administration (rfMDA).* Reactive focal drug administrations at household level were presumed to continue as well, but with decreasing costs over time (by 20% till 2020 and by 50% onwards) given the potential know-how synergies inherent to the intervention (e.g. on its first year, personnel and transport needs were overestimated, so it was planned to reduce fieldworkers from 24 to 15 and employ bicycles -1 per fieldworker- instead of 3 cars in the future).
- *Malaria incidence and case management costs.* We assumed that malaria incidence, both under the Magude project and control scenario, would remain the same as the levels observed (or expected) by June 2018 – according to study impact estimates [1] – but adjusted year to year taking into consideration the Mozambican population growth rates till 2030 (average of per year). Unit treatment costs, both for inpatient and outpatients, were assumed as invariant.

All costs projections were expressed in constant 2015 US$ and were depreciated, annualized, inflated – using the average expected inflation rate [2] in the country- and discounted employing the parameters from table S1 and the formulas described above, considering a timespan until 2030.

**Reference List**

1. Galatas B, Saúte F, Martí-Soler H, Montañà J, Guinovart C, Munguambe H, et al. The Magude project: a before-after study aiming to eliminate malaria in southern Mozambique. Manuscript under review (PLos Med). 2020.

2. WB. The World Bank. The World Bank Open Data 2018.
